# Competitive Propagation Laws of Hydraulic Fractures in Multiple Thin Coal Seams

**DOI:** 10.1021/acsomega.5c13619

**Published:** 2026-04-13

**Authors:** Hui Xiao, Tianxi He, Hongsen Wang, Chang Yuan

**Affiliations:** School of Petroleum Engineering, 66564 Chongqing University of Science and Technology, Chongqing 401331, China

## Abstract

This study investigates nonuniform hydraulic fracture propagation in vertically stacked thin coal seams with small interlayer spacing. A discrete element-based numerical model incorporating dynamic interlayer flow allocation was developed to simulate competitive fracture behavior. Key findings include: (1) As interlayer stress difference increases (from 1.5 to 4 MPa), fractures shift from interlayer penetration to coal seam propagation, with main seam fracture length growth dropping sharply (from 18.37–20.73% to 2.59–6.07%) and nonuniform propagation coefficient rising (from 0.06 to 0.33). When the leakoff coefficient increases (from 0.0005 to 0.0030 m/min^0.5^), the thinnest seam’s fracture length reduces by 36.70% while its flow proportion increases (from 12 to 33%). (2) Flow allocation trends: higher stress difference favors thick seams; elevated leakoff benefits thin seams; increased thickness proportion advantages thick seams (11#flow: from 38 to 60%). (3) Due to significant physical property differences between coal seams and roof/floor rocks (coal elastic modulus: 5–12 GPa vs roof/floor elastic modulus: 25 GPa), fractures primarily extend along coal seams with strong roof/floor barrier effects (coal flow >90%). (4) Increased coal seam thickness proportion enhances the competitive advantage of thick seams, with the nonuniform propagation coefficient remaining at 0.28 and thin seam propagation being suppressed. This work provides a theoretical basis for optimizing multithin-layer coal seam fracturing. This work provides a theoretical basis for optimizing multithin-layer coal seam fracturing.

## Graphical abstract

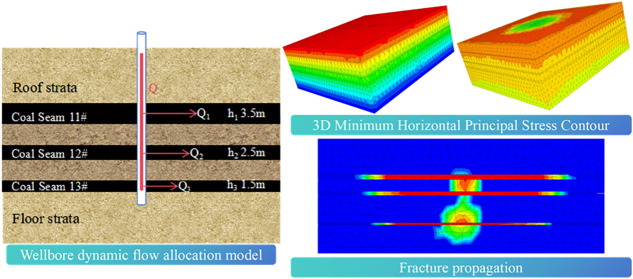

## 1 Introduction

Multiple thin coal seams are vertically stacked, featuring numerous layers (typically 5–15 or more), thin individual layers (single layer thickness mostly 0.5–1.5 m), and small interlayer spacing (mostly within 2–10 m), and exhibit significant cleat development characteristics. These coal seams show large lateral lithological variations. They also have significant differences in key parameters including interlayer stress difference, permeability and elastic modulus.^1^ This geological characteristic makes hydraulic fractures highly prone to interlayer barrier effects during propagation. In practical engineering applications, hydraulic fracturing for multiple thin coal seams typically employs staged fracturing technology. This involves dividing the target fracturing zone into 2–3 fracturing stages (each stage total thickness ∼ 10–30 m) based on the geological characteristics of the coal seam group. Each fracturing stage may be further subdivided into 2–5 sublayers (individual sublayer thickness ∼ 0.5–3 m) for targeted stimulation. However, due to differences in the physical properties (e.g., stress, leakoff coefficient, thickness) of each sublayer, significant competitive propagation for flow occurs among the sublayers during fracturing fluid injection,^1^ easily leading to uneven fluid distribution and insufficient stimulation of some sublayers. Therefore, studying the competitive propagation laws of hydraulic fractures in multiple thin coal seams is key to optimizing fracturing design and improving stimulation effectiveness for such reservoirs. Recent numerical studies have further highlighted the complexity of vertical-lateral competitive propagation in multilayered formations and the potential of novel well architectures like fishbone multilateral wells in deep coal seams.^2,3^

Regarding dynamic flow allocation, Weng et al.^4^ established a complex hydraulic fracture model to simulate the propagation of a fracture network in naturally fractured formations, accounting for dynamic fluid distribution among multiple branches. McClure et al.^5^ used a discrete fracture network approach to simulate dynamic flow allocation in complex fracture systems, highlighting the importance of perforation erosion. Hamdia et al.^6^ presented an analytical solution for fluid distribution among multiple fractures, considering wellbore hydraulics and near-wellbore effects. Peirce and Detournay^7^ developed an implicit level set algorithm for simulating simultaneous propagation of multiple hydraulic fractures, capturing the competitive fluid allocation. Li et al.^8^ established a DEM coupling model to simulate shale oil reservoir hydraulic fracturing, considering perforation erosion and flow allocation, revealing that low interfacial angles favor vertical propagation, while high interfacial angles and bedding planes restrict vertical propagation through shear failure, and natural fractures exacerbate propagation nonuniformity. Zhao et al.^9^ established a multifracture fluid–solid coupling model; simulations show that increasing cluster number intensifies inner-cluster suppression (fracture width/length/flow decrease), and increasing cluster spacing can reduce stress interference and improve inner-cluster flow allocation.

Regarding multithin-layer fracture propagation simulation, Jeffrey et al.^10^ investigated the vertical propagation of hydraulic fractures in layered formations using a 3D numerical model. Wu and Olson^11^ developed a fully coupled model for simulating multiple fracture propagation in horizontal wells, addressing the stress interference effects. Lecampion et al.^12^ presented a review of numerical methods for hydraulic fracturing, covering various approaches for modeling fracture propagation in complex geological settings. Olson and Dahi-Taleghani^13^ used a hybrid finite-discrete element method to simulate fracture propagation in naturally fractured reservoirs, capturing the interaction between hydraulic and natural fractures. Fisher and Warpinski^14^ provided a foundational review of hydraulic fracture height containment, emphasizing the critical role of stress contrasts and layer interfaces, which is directly relevant to the interlayer propagation in multithin-coal-seam systems. Feng et al.^15^ applied the Material Point Method (MPM) to simulate shale reservoir fracturing; results matched well with actual stress fields and microseismic data, which were used to optimize well trajectory azimuth (angle to maximum horizontal principal stress) to increase stimulated area. Wu^16^ used elastoplastic fracture mechanics finite element method (ANSYS) to simulate fracturing effects near boreholes, analyzing the influence of well pressure, reservoir depth, perforation length, and fracture development degree on formation stress. Cong et al.^17^ based on the 3D discrete lattice method, used Xsite software to establish a fracture height propagation model, simulating the fracture interlayer propagation process in layered formations under the combined action of in situ stress, rock properties, and operational parameters. Liu et al.^18^ used the finite element method to establish a 3D fluid–solid coupling model, studying the propagation laws of multiple vertical fractures at different depths of a vertical wellbore. Numerical simulation results indicate connectivity phenomena between fractures in adjacent layers; the possibility of multifracture connectivity increases when interlayer stress is low and Young’s modulus is high. Li et al.^19^ used the combined finite-discrete element method to construct a 3D simulation model for multicluster fracturing, studying the propagation mechanism of multicluster hydraulic fractures in deep shale. Wu et al.^20^ established a 3D multicluster reservoir fracturing model based on cohesive element modeling, analyzing the propagation laws of artificial fractures and related quantitative parameter laws in a reservoir in Southwest China under the influence of various parameters.

Current research on hydraulic fracturing in multithin-seam coalbeds has revealed the influence of parameters such as stress differential and filtrate loss coefficient. However, the control exerted by the unique fracture systems inherent to coal reservoirs—such as cleavage planes and natural fractures—on hydraulic fracture propagation requires further investigation in light of the latest findings. Li et al.^21^ systematically reviewed the propagation characteristics of hydraulic fractures in coalbed methane reservoirs, indicating that the coal’s joint network (both planar and end joints) directly controls the initiation orientation and propagation path of hydraulic fractures by altering stress distribution and fluid flow pathways. When encountering high-density fractures, hydraulic fractures tend to branch rather than penetrate, resulting in reduced effective fracture length but expanded connectivity. This conclusion provides key theoretical support for understanding fracture propagation differences between coal reservoirs and conventional sandstone reservoirs. Changbao et al.^22^ further validated through laboratory physical simulation tests that the orientation (dip angle, density) of natural fractures exhibits a threshold effect on coal hydraulic fracturing: When the angle between natural fractures and hydraulic fractures is less than 30°, hydraulic fractures readily propagate along natural fractures, triggering “deflection-through” phenomena to form complex fracture networks. Conversely, when the angle exceeds 60°, natural fractures exhibit a “barrier effect,” inhibiting longitudinal penetration of hydraulic fractures.

Beyond the methodologies cited above, the integration of numerical models with geophysical monitoring and advanced statistical approaches has gained significant traction for validation and optimization purposes. For instance, Bosikov et al.^23^ demonstrated the enhanced reliability of modeling fracture network growth by incorporating geophysical methods, allowing for a visual assessment of permeability enhancement in geological masses. Similarly, the application of three-dimensional statistical models, as shown by Brigida et al.,^24^ can identify local extremes for optimizing process productivity. These studies underscore that numerical models achieve greater robustness and environmental relevance when calibrated against geophysical data and real in situ measurements, paving the way for more sustainable resource extraction methods.

It is worth comparing the fundamental approaches of these methods. The Finite Element Method (FEM), employed in many of the cited studies, is highly effective for solving problems involving continuous media deformation and fluid–solid coupling, based on the assumption of material continuity. In contrast, the Discrete Element Method (DEM) adopted in this study does not rely on this continuity assumption. Instead, it explicitly represents a rock mass as an assemblage of discrete blocks, allowing for a more natural simulation of fracture initiation and propagation along pre-existing or newly generated discontinuities, as well as the subsequent complex interactions between blocks. This capability is particularly advantageous for modeling the competitive propagation of hydraulic fractures in coal seams, which are characterized by well-developed cleats and significant heterogeneity, where the fracture path is dominantly controlled by the interaction and failure of pre-existing discontinuities.

Currently, significant achievements have been made in research on multilayer fracturing for low-permeability sandstone and shale reservoirs, forming a relatively systematic theoretical system regarding competitive fracture propagation laws and dynamic flow allocation. However, coal seams, as typical organic sedimentary rocks, exhibit significant differences in bedding structure, mechanical properties, and stress environment compared to sandstone. For instance, coal seams exhibit stronger heterogeneity, anisotropy, lower rock strength, and more complex interlayer stress differences and cleat development characteristics. These differences make hydraulic fracturing models and design methods for sandstone reservoirs difficult to apply directly to coal seam development. This model primarily focuses on the competitive propagation of fractures along vertical cross sections. Consequently, it places particular emphasis on the controlling effect of the difference in the minimum horizontal principal stress (σ_hmin_) between the coal seams and their immediate roof and floor strata on fracture height. This simplified assumption is widely adopted in engineering models for analyzing fracture height containment issues in multilayer systems. Therefore, targeting the geomechanical characteristics of coal seams, this paper describes fluid behavior using the continuity equation and non-Newtonian fluid flow equations, implements dynamic wellbore flow allocation based on Darcy’s law, and establishes a discrete element simulation model for coal-rock fracture propagation considering dynamic flow allocation. This model is used to study the propagation, competition, and connectivity mechanisms of hydraulic fractures in multiple thin coal seams, providing theoretical support and technical guidance for efficient coalbed methane development.The complexity of these mechanisms is underscored by recent works exploring intelligent prediction of fracture paths in coal measure strata^25^ and the development of sophisticated two-level flow distribution models to understand nonuniform propagation.^26^

## 2 Coal-Rock Fracture Propagation Model Considering Dynamic Flow Allocation

### 2.1 Mathematical Model for Fracture Propagation

#### 2.1.1 Block Motion and Deformation

Within the 3DEC discrete element framework,^27^ the propagation of hydraulic fractures is the result of the dynamic evolution of a block system under the combined action of fluid pressure, in situ stress, and constitutive relationships. The core mechanisms are described by the following equations:

The translation and rotation of each discrete block obey Newton’s second law:

The translational equation is given by

1 $$m_{i}\frac{d^{2}u_{i}}{dt^{2}}=F_{i}^{\mathrm{tot}\mathrm{al}}$$

The rotational equation is given by

2 $$I_{i}\frac{d^{2}\theta_{i}}{dt^{2}}=M_{i}^{\mathrm{tot}\mathrm{al}}$$

Where *m*_*i*_ is the mass of block i, *u*_*i*_ is the translational displacement vector of the block, *F*_*i*_^total^ is the resultant force acting on the block (including body force, contact force, and fluid pressure); *I*_*i*_ is the moment of inertia of the block, θ_*i*_ is the rotational angle vector, *M*_*i*_^total^ is the resultant moment acting on the block.

The internal stress–strain relationship of deformable blocks satisfies the constitutive equation

3 σ=C:ε

Where σ is the stress tensor, ε is the strain tensor, and *C* is the stiffness tensor (determined by material properties).

#### 2.1.2 Contact Mechanics and Fracture Characterization

Contact forces between blocks are calculated through contact constitutive models, focusing on normal and tangential behaviors:

Normal force (linear elastic model)

4 $$F_{n}=k_{n}\cdot U_{n}$$

Where *k*_n_ is the normal stiffness, *U*_n_ is the normal relative displacement (contact pressure is generated when *U*_n_ > gap, contact enters a tension state when *F*_*n*_ < −*T*, and *T* is the contact tensile strength).

Tangential force (Coulomb slip model)

5 $$\Delta F_{s}=-k_{s}\cdot \Delta U_{s}$$

Where *k*_s_ is the tangential stiffness, Δ*U*_s_ is the increment of tangential relative displacement, friction satisfies |*F*_s_| ≤ *c* + *F*_n_·tanϕ (*c* is cohesion, ϕ is friction angle), and sliding occurs when this value is exceeded.

Fractures are characterized by contact states: open or sliding contacts constitute fractures, and their location, orientation, and aperture (dynamically calculated from *U*_n_) evolve in real-time with block motion.

#### 2.1.3 Fluid Flow

Fluid flows within the network of open fractures. The core equations are as follows:

The cubic law defines the flow rate through a single fracture as

6 $$q=-\frac{w^{3}}{12\mu }\left(\nabla p-\rho_{f}g\right)$$

Where *w* is the fracture hydraulic aperture, μ is the fluid viscosity, ∇*p* is the pressure gradient, ρ_*f*_ is the fluid density, and *g* is the gravitational acceleration. Continuity equation (fracture network nodes):

7 $$\sum \left(q_{\mathrm{in}}\right)-\sum \left(q_{\mathrm{out}}\right)+Q_{\mathrm{source}}-Q_{\mathrm{leakoff}}=\frac{d\left(V_{f}\cdot \rho_{f}\right)}{\mathrm{dt}}$$

Where *q*_in_/*q*_out_ are inflow/outflow rates, *Q*_source_ is the injection source term, *Q*_leakoff_ is the matrix leakoff volume, and *V_f_* is the fluid volume controlled by the node, the right side is the rate of change of fluid mass over time^28^ (considering compressibility and aperture change).

Non-Newtonian fluid modification (power-law fluid): The viscosity μ in the cubic law needs to be replaced by the effective viscosity μ_eff_ *(*γ̇*)* dependent on shear rate. Shear rate γ can be estimated from flow velocity and aperture within the fracture τ = *k·*γ̇*n*; eq 6 is modified to

8 $$q=-\frac{w^{2}}{f\left(n\right)}{\left(\frac{w\cdot \left|\nabla p\right|}{k}\right)}^{1-n/n}\cdot \mathrm{sign}\left(\nabla p\right)$$

Where τ is the shear stress, *k* is the consistency coefficient, *n* is the power-law index, and *f*(*n*) is a correction factor related to *n*.

#### 2.1.4 Hydraulic Fracturing Driving and Propagation Criteria

##### 2.1.4.1 Main Driving Mechanism

Injected fluid increases the pressure *p*_*f*_ within the fracture, reducing the effective normal stress acting on the fracture face:^29–31^

9 $$\sigma_{n}^{\mathrm{eff}}=\sigma_{n}-p_{f}$$

##### 2.1.4.2 Contact Failure Criteria

Fracture propagation is primarily achieved through the failure of contact points between discrete blocks. The tensile and shear failure criteria employed in this model are commonly used in the discrete element method to simulate the tensile and shear failure of rock masses. This approach can effectively characterize the entire process of fracture initiation, propagation, and coalescence. The specific criteria include:

Tensile failure: When the effective normal stress σ_*n*_^eff^ at the contact point exceeds the contact tensile strength T:^32^

10 $$\sigma_{n}^{\mathrm{eff}}>Τ$$

Shear failure (Coulomb criterion): When the tangential stress exceeds the shear strength:^33^

11 $$\left|\tau \right|>c+\sigma_{n}^{\mathrm{eff}}\cdot \tan\varphi$$

After failure, contact strength parameters (*c*, ϕ, and *T*) are significantly reduced, and relative block motion intensifies, forming new fracture propagation.

### 2.2 Wellbore Dynamic Flow Allocation Criterion

Assume the multithin-layer coal seam consists of n thin layers; each layer can be regarded as an independent flow unit. For the *i*th thin layer, its flow rate Q_i_ satisfies Darcy’s law:^34^

12 $$Q_{i}=\frac{k_{i}A_{i}\Delta p_{i}}{\mu_{i}L_{i}}$$

Where *Q* is the flow rate (m^3^/s); *k* is the permeability of the porous medium (m^2^); *A* is the cross-sectional area of fluid flow (m^2^); Δ*p* is the pressure drop resulting from wellbore friction and perforation friction (Pa); μ is the fluid viscosity (Pa·s); *L* is the length of the fluid flow path (m).

To isolate and highlight the influence of key parameters of interest (i.e., permeability and thickness) on competitive propagation, the following simplifying assumptions are adopted:

(1) Identical fluid properties: The viscosity of the fracturing fluid in each thin layer is assumed identical, μ*_i_* = μ (*i* = 1,2,···,*n*).
(2) Identical pressure difference: During staged fracturing, the pressure difference Δ*p_i_* = Δ*p* (*i* = 1,2,···,*n*) across each thin layer is the same. The external pressure acting on each layer under the same fracturing operation is essentially consistent, leading to this identical pressure difference.
(3) Identical fracture width: The fracture width (*w*) is assumed to be identical for all thin layers, *w_i_* = *w* (*i* = 1,2,···,*n*). This is a simplification that allows us to focus on the competition driven by permeability and thickness variations.

It should be noted that the assumptions of identical fluid viscosity, pressure difference, and fracture width across all layers are adopted to isolate and emphasize the dominant controlling factors (e.g., permeability and thickness) on competitive fracture propagation. While these simplifications facilitate a clearer understanding of the fundamental mechanisms, they may not fully capture the complexities encountered in field applications, such as variations in fluid rheology, differences in perforation friction, and layer-specific pressure drops. Therefore, the quantitative results derived from this model should be interpreted within the context of these assumptions. Future work could incorporate more realistic representations of fluid and wellbore dynamics to enhance the model’s predictive capability for field-scale engineering design.

Under these assumptions, the flow rate formula for the *i* thin layer simplifies to

13 $$Q_{i}=\frac{k_{i}wh_{i}\Delta p}{\mu L_{i}}$$

The schematic of wellbore dynamic flow allocation is shown in Figure 1.

**Figure 1:**
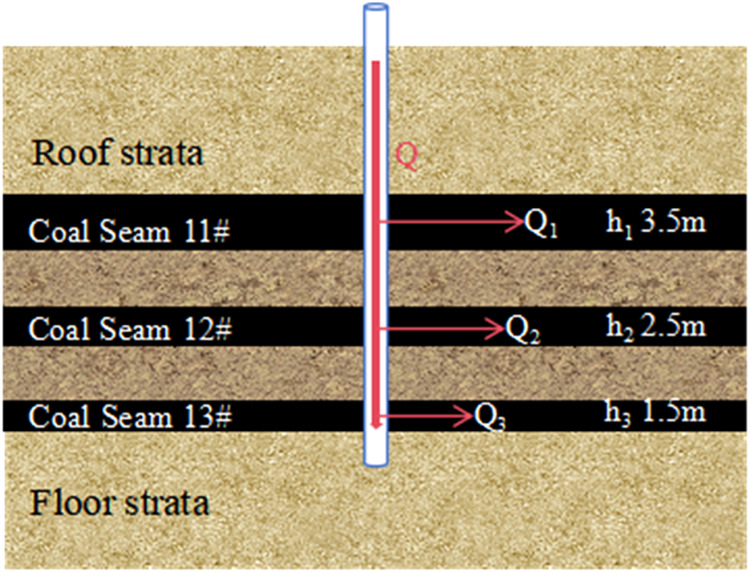
Schematic of wellbore dynamic flow allocation model.

The total flow rate *Q*_total_ is the sum of the flow rates of all thin layers

14 $$Q_{\mathrm{total}}=\sum_{i=1}^{n}Q_{i}=\sum_{i=1}^{n}\frac{k_{i}wh_{i}\Delta p}{\mu L_{i}}=\frac{w\Delta p}{\mu }\sum_{i=1}^{n}\frac{k_{i}h_{i}}{L_{i}}$$

Then the proportion *f*_*i*_ of the flow rate of the *i*th thin layer to the total flow rate is

15 $$f_{i}=\frac{Q_{i}}{Q_{z}}=\frac{\frac{k_{i}wh_{i}\Delta p}{\mu L_{i}}}{\frac{w\Delta p}{\mu }\sum_{j=1}^{n}\frac{k_{j}h_{j}}{L_{j}}}=\frac{\frac{k_{i}h_{i}}{L_{i}}}{\sum_{j=1}^{n}\frac{k_{j}h_{j}}{L_{j}}}$$

### 2.3 Model Solution

Based on the 3DEC discrete element principle, the model^35^ is solved using an explicit time-stepping method. We adopt the DEM instead of continuum-based methods (e.g., FEM) because DEM excels at simulating discontinuous mechanical behaviors. This method is especially suitable for capturing complex processes such as crack initiation, propagation and coalescence. It also accurately simulates the dynamic interaction between fluid pressure and block movement—both are core issues of competitive fracture growth in layered coal masses. By coupling block mechanical motion^36^ with fracture network fluid flow, dynamic simulation of hydraulic fracture propagation in multithin-layer coal-rock masses is achieved. The solution process centers on ″mechanics-fluid″ coupled calculations, with specific steps as follows:

Initialization Setup: Construct the discrete block system; assign physical and mechanical parameters to blocks (density ρ, elastic modulus *E*, Poisson’s ratio ν) and contact properties (normal stiffness *k*_n_, tangential stiffness *k*_s_, cohesion μ, friction angle ϕ, tensile strength T); Apply the initial in situ stress field: The far-field in situ stresses, including the vertical stress (σ_v_), maximum horizontal principal stress (σ_H_), and minimum horizontal principal stress (σ_h_), are applied as normal stress boundary conditions on the outer boundaries of the model. An equilibrium calculation is then performed. The 3DEC solver iteratively adjusts the contact forces between blocks until the resultant force and moment on every block are zero, ensuring the entire block system reaches a state of static equilibrium under the initial conditions; Define fracture network fluid parameters: set the relationship between fracture aperture and conductivity (e.g., w^3^/12), fluid properties (viscosity μ, density *ρf*, power-law parameters *k*/*n*), matrix leakoff coefficient, and injection boundary conditions (injection point location, flow rate *Q*_s_, or pressure difference Δ*p*); initialize time *t* = 0, and set time step size Δ*t* (determined based on block stability and computational efficiency).

Main Solution Loop (per time step Δ*t*): Fluid Flow Calculation (executed every N mechanics steps): Based on the current aperture w of the fracture network (obtained from block contact state), calculate the conductivity κ = *w* ^3^/12 for each fracture; Assemble the mass conservation eq (eq 7) for fracture network nodes, coupled with the cubic law (eq 6), to solve for fluid pressure distribution *p_f_*; calculate leakoff volume Q_leakoff_ based on matrix permeability and pressure gradient; update fluid volume within fractures.

Mechanics Calculation: ① Contact Force Calculation: Based on current block displacement *u_i_* and rotation θ*_i_*, combined with the contact constitutive model (normal force eq 4, tangential force eq 5), calculate normal force *F*_n_ and tangential force *F*_s_ for all contact points. ② Fluid Pressure Loading: Apply the solved fracture internal pressure *p_f_* as a surface force to the block boundaries corresponding to fracture faces, participating in block force equilibrium. ③ Block Motion Equation Integration: For each block, based on Newton’s second law (eqs 1, 2), calculate resultant force and moment, and integrate to obtain acceleration, velocity, and new displacement and rotation. ④ Contact Failure Judgment: Based on effective stress (eq 9) and failure criteria (eqs 10, 11), determine if tensile or shear failure occurs at contact points. If failure occurs, reduce contact strength parameters and mark new fracture formation.

State Update and Flow Allocation: Update block positions, contact states, and fracture apertures (calculated from normal relative displacement *U*_n_), correct fracture network topology; based on the flow allocation criterion in Section 2.2, calculate the flow allocation proportion ***f***_***i***_ for each thin layer using eq 14, and apply flow rate *Q_i_* as source term *Q*_source_ to corresponding fracture network nodes. Update the time step; if the preset simulation time is not reached, return to the fluid flow calculation step; if termination conditions are met, output fracture morphology (location, aperture, and connectivity), fluid pressure field, block displacement, and flow allocation results. This solution process realizes dynamic fluid-mechanical coupling via explicit time stepping. It retains the DEM’s advantage in detailed characterization of block motion and contact failure, and accurately simulates fluid flow behavior in fracture networks through the flow model.^37^ Ultimately, it dynamically correlates wellbore flow rate with fracture propagation by the proposed flow allocation criterion. Finally, it dynamically links wellbore flow rate with fracture propagation through the flow allocation criterion. The specific steps are shown in Figure 2.

**Figure 2:**
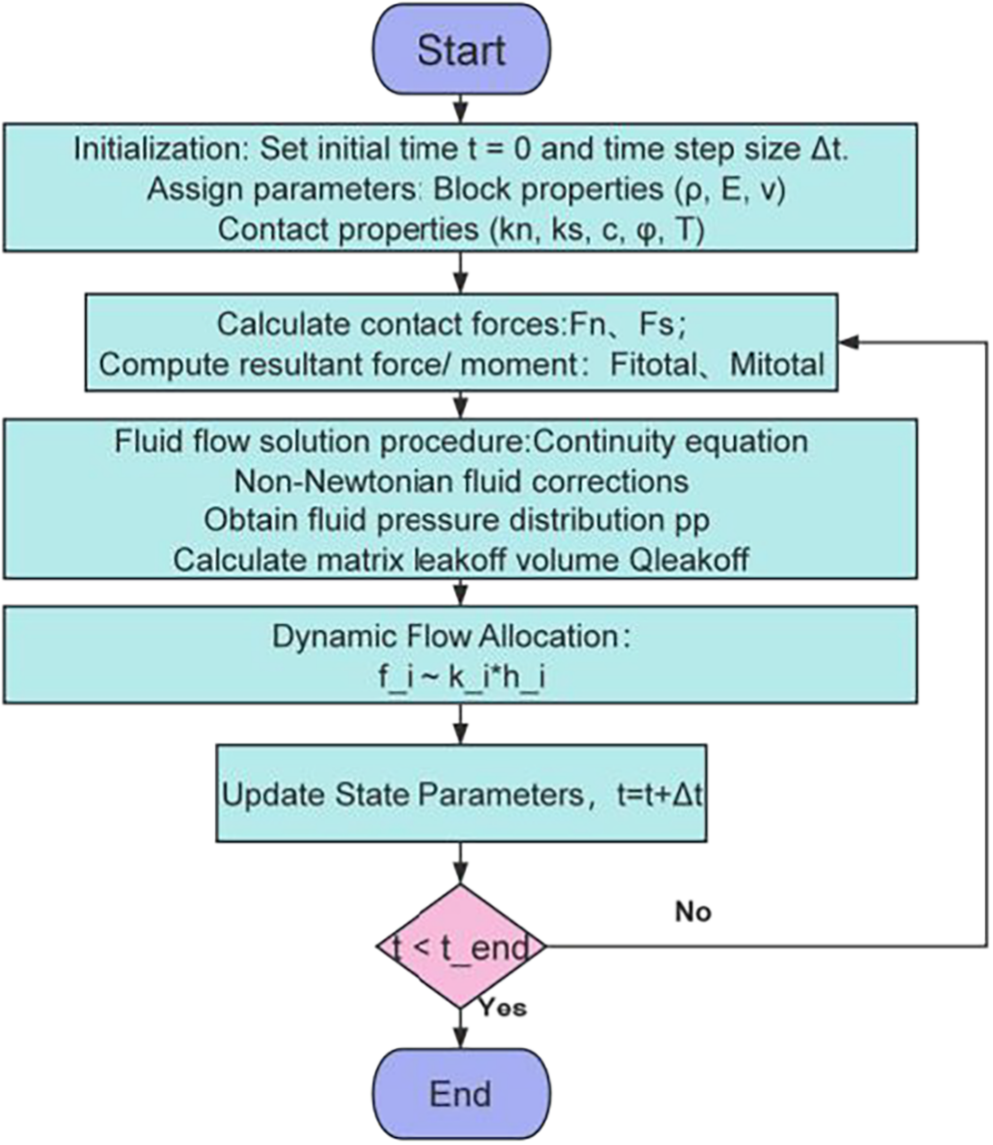
Solution procedure for the fracture propagation model.

Through the above numerical solution method, detailed spatiotemporal distribution laws of fracture geometric parameters and fluid flow parameters were obtained, providing strong support for further analysis of influencing factors and mechanisms of fracture propagation.

## 3 Model Establishment and Validation

### 3.1 Geometric Model

Based on coal seam data from the Longtan Formation in western Guizhou, China, rock mechanical parameters are shown in Table 1 and fluid properties in Table 2. A numerical simulation model was established according to actual coal seam characteristics, including three coal seams: No. 11#, No. 12#, and No. 13#. Model dimensions: 200 m in the direction of maximum principal stress, 200 m in the direction of minimum principal stress, and 80 m in the vertical stress direction, as shown in Figure 3. The rock mechanical properties of the three coal seams are identical. Outside the coal seams are the roof and floor strata, whose data are also identical.

**Table 1:** Geometric Dimensions and Material Parameters of the Numerical Model

coal seam		roof/floor strata		fluid parameters	
input parameter	value	input parameter	value	input parameter	value
elastic modulus E (GPa)	5–12	elastic modulus E (GPa)	25	fluid bulk modulus K (MPa)	3
Poisson’s ratio μ	0.24–0.27	Poisson’s ratio μ	0.2	fluid density (kg/m^3^)	1000
rock density (kg/m^3^)	1200	rock density (kg/m^3^)	2590	fluid viscosity η (Pa·s)	0.0015
leakoff coefficient (m/min^0.5^)	5 × 10^–4^	leakoff coefficient (m/min^0.5^)	1 × 10^–6^		
coal seam thickness (m)				roof/floor thickness (m)	
11#		3.5		35, 7, 16, 14.5	
12#		2.5			
13#		1.5			

**Figure 3:**
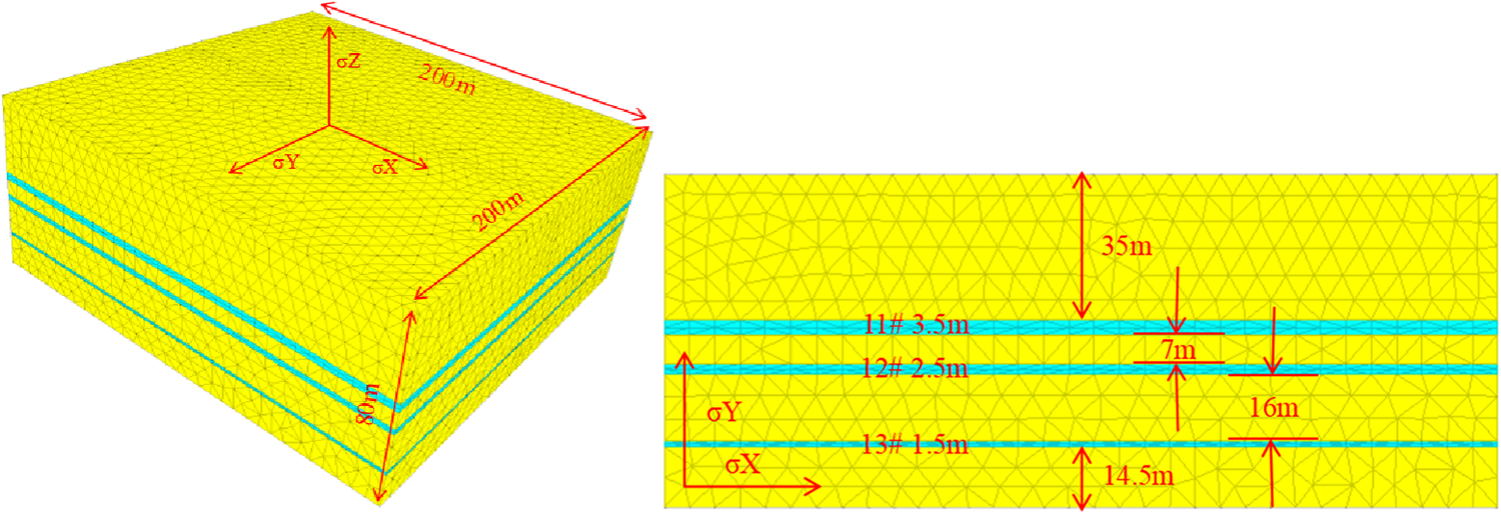
Schematic diagram of the 3D coalbed methane hydraulic fracture propagation model.

The model dimensions were chosen to be significantly larger than the expected fracture propagation range to minimize boundary effects. The boundary conditions were set as stress boundaries to simulate the far-field in situ stress state. Specifically, constant normal stresses corresponding to the three principal in situ stresses were applied to the model’s outer boundaries. For the parameter studies, the vertical stress (σ_v_) and the maximum horizontal principal stress (σ_H_) were maintained at a constant ratio to the minimum horizontal principal stress (σ_h_), while the value of σh for the coal seams was varied relative to the roof and floor to create the desired interlayer stress contrast. The established initial stress field is visually represented by the 3D contour plot of the minimum horizontal principal stress before fracturing (Figure 4a), while Figure 4b clearly captures the redistributed stress field after the fracturing treatment.This approach allows us to isolate the effect of the minimum horizontal stress difference, which is the primary controller of fracture height containment, on competitive propagation.

**Figure 4:**
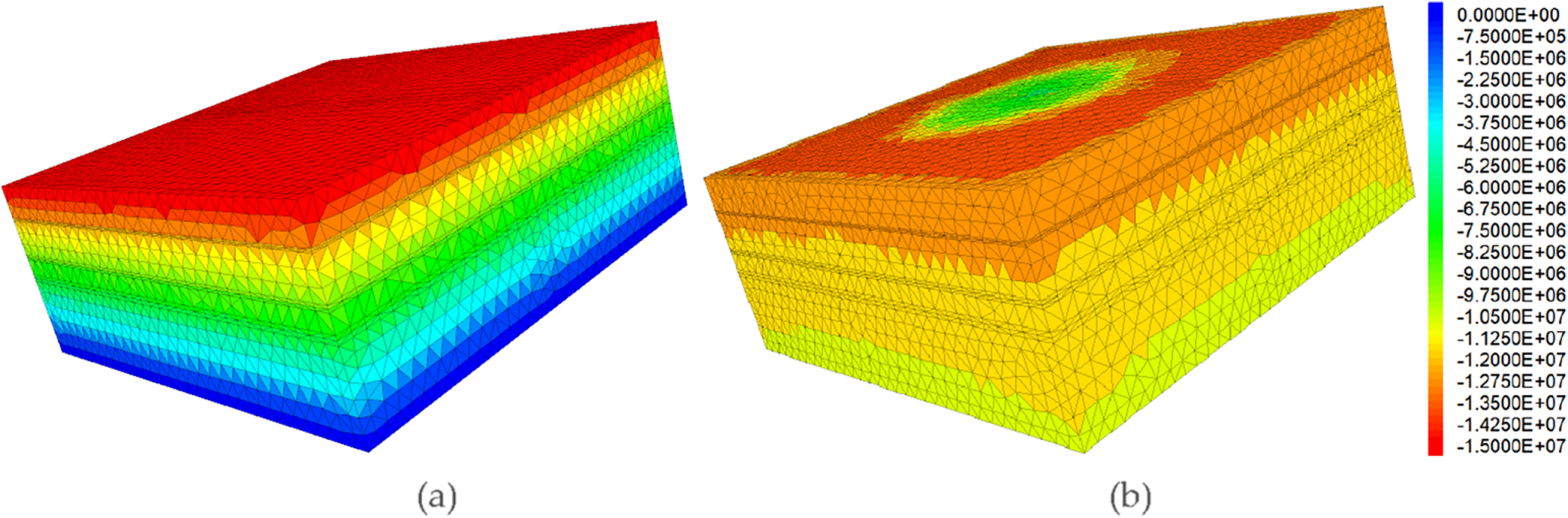
3D minimum horizontal principal stress contour of hydraulic fracture propagation in coalbed methane reservoirs. (a) Before fracturing; (b) after fracturing.

It is noteworthy that coal contains well-developed cleats (as shown in Figure 5), which constitute inherent weak planes and significantly influence its mechanical and seepage behavior.

**Figure 5:**
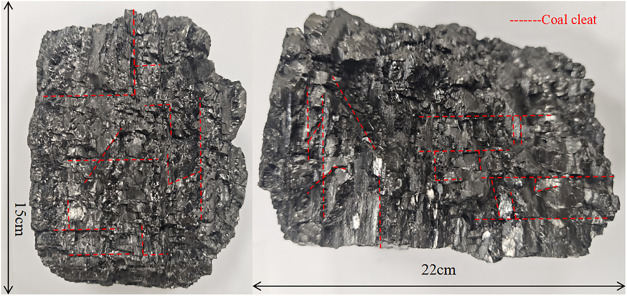
Schematic diagram of fracture development in coal seam rock blocks of the Longtan Formation, Guizhou Province, China.

As illustrated in Figure 6a, face cleats and butt cleats are not perfectly orthogonal, and cleat spacing and length vary considerably across different coal seams—ranging from millimeters to tens of meters. These structural characteristics, along with other weak planes, can affect hydraulic fracture propagation in complex ways and pose considerable computational challenges in discrete element modeling. Therefore, appropriate model simplification is necessary. In this study, the discrete element model focuses on simulating the competitive propagation of hydraulic fractures at a larger interlayer scale, emphasizing the controlling effect of the mechanical contrast between coal and roof/floor rocks. The primary fracture path is governed by this coal-rock interface contrast and the in situ stress state. The initiation and propagation of new fractures driven by fluid pressure, as well as shear slip along pre-existing discontinuities, are inherently captured within the DEM framework through contact failure. Although the actual cleat system is highly complex, the model’s representation of discrete fracture networks effectively captures key mechanical behaviors relevant to the scale of interest. This simplification, as reflected in the schematic of coal modeling shown in Figure 5b, is considered reasonable for the primary objectives of this study.

**Figure 6:**
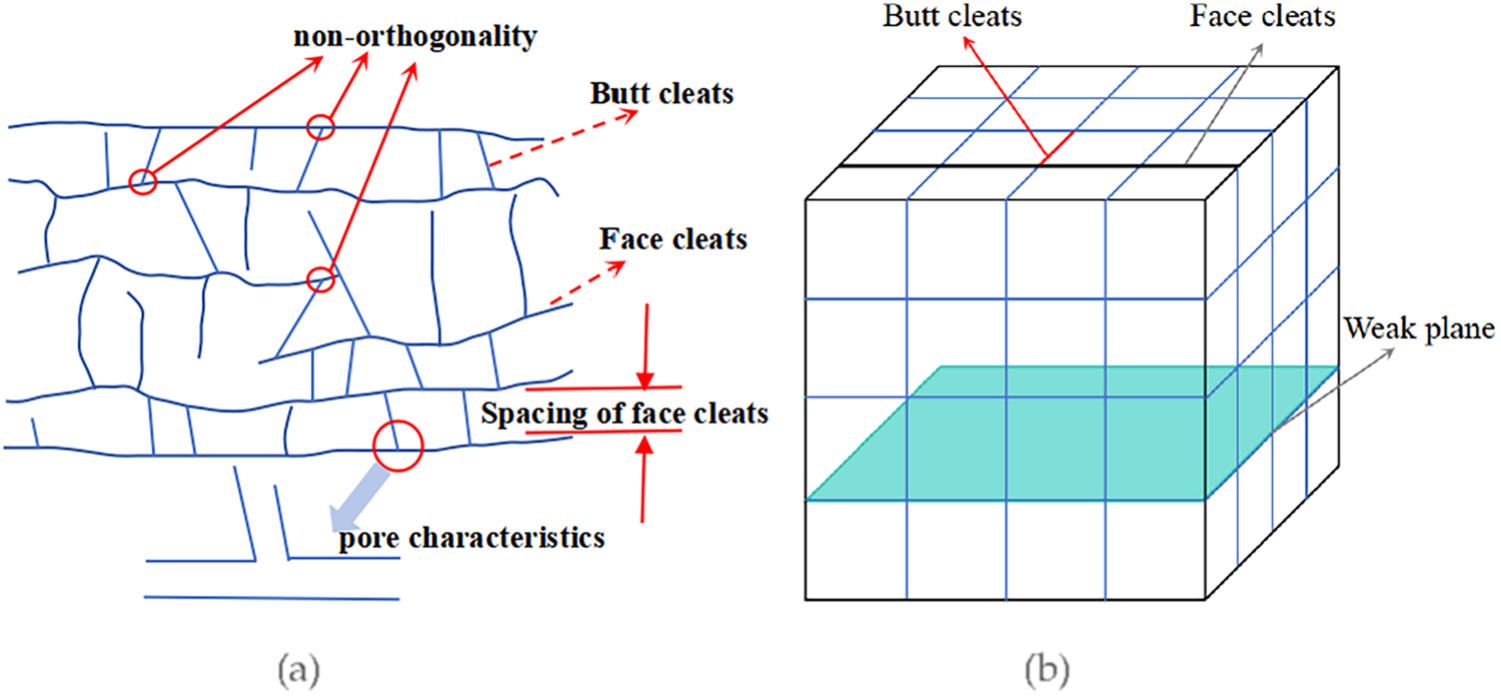
(a) Schematic diagram of coal block cleats. (b) Schematic diagram of coal mass modeling.

### 3.2 Model Validation

Figure 7 compares the fluid injection pressure from the numerical simulation model with actual field fracturing treatment pressure. The high agreement between numerical simulation results and actual field fracturing treatment pressure strongly corroborates the reliability of the model.

**Figure 7:**
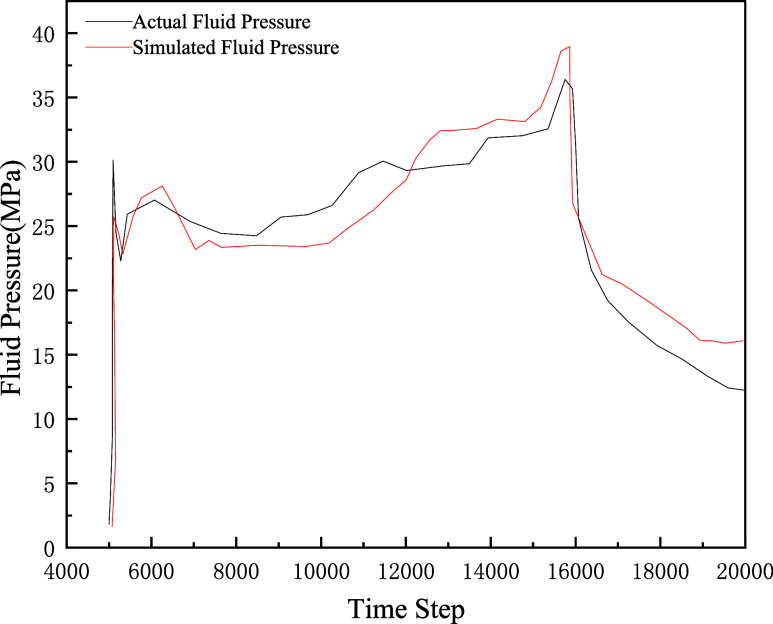
Pore fluid pressure variations at different locations.

To improve grid structure stability and reduce impact on simulation results, encrypted grid division was adopted. Comparing microseismic monitoring and simulation results shows that grid division has almost no effect on the results.

To further validate the model’s accuracy in predicting fracture geometry, the simulated fracture lengths were compared with field microseismic monitoring results. As shown in Figure 8 and Table 2, the microseismic monitoring indicated fracture lengths of 179, 172, and 170 m for seams 11#, 12#, and 13# respectively, while the simulation yielded 168, 163, and 161 m, with all difference ratios below 6.15%. This close alignment further confirms the model’s reliability in capturing fracture propagation characteristics.

**Figure 8:**
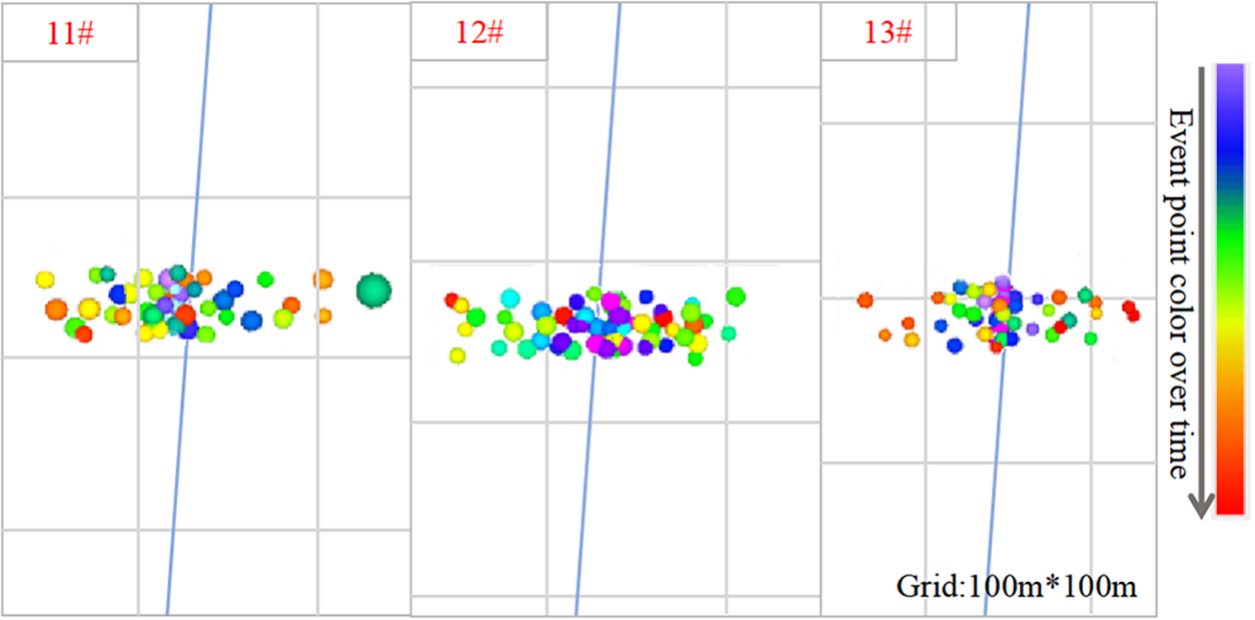
Microseismic fracture monitoring results for coal seams 11#, 12#, and 13# in the Longtan Formation.

**Table 2:** Geometric Dimensions and Material Parameters of the Numerical Model

	microseismic monitoring	simulation	difference ratio (%)
comparison parameters	fracture length (m)		
11#	179	168	6.15
12#	172	163	5.23
13#	170	161	5.29

## 4 Results and Discussion

### 4.1 Interlayer Stress Difference

Coal seams and adjacent roof/floor strata have obvious differences in lithology and physical properties (e.g., permeability, elastic modulus). This interlayer heterogeneity leads to a distinct selectivity in fracture propagation: fractures tend to extend within coal seams instead of penetrating interlayers. Consequently, fluid primarily flows within relatively independent thin-layer networks inside the coal seams. In this context, fracture length becomes a key parameter determining fracture communication range and seepage channel connectivity, directly controlling the reservoir volume covered by a single fracture and the competitive relationships between multiple fractures. Therefore, based on the ″intralayer dominance, interlayer segmentation″ seepage characteristic of coal seams, prioritizing fracture length as the core variable can more efficiently characterize the flow allocation rules and competition mechanisms of multithin-layer fracture systems.

Figure 9 shows simulation results under a displacement rate of 10 m^3^/min for different stress differences (difference in minimum horizontal principal stress between coal seam and roof/floor).^38^ As the interlayer stress difference increases, the fracture’s ability to penetrate interlayers continuously weakens. The growth trend of fracture length gradually shifts from significant increase to being suppressed. A low-stress-difference environment is more conducive for fractures to penetrate interlayers.

**Figure 9:**
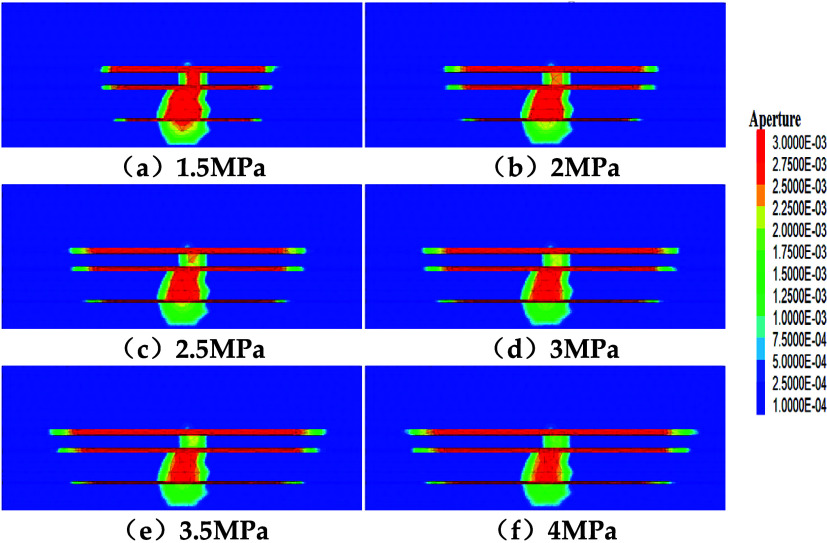
Fracture propagation under different stress differences (a–f).

Analyzing fracture length: When the interlayer stress difference is small (1.5–2 MPa), fractures possess a stronger ability to penetrate interlayers, and fracture length growth is relatively obvious. Specifically, No. 11# seam fracture length increased by 20.73%, No. 12# by 19.52%, and No. 13# by 18.37%. When the interlayer stress difference reaches a high level (3.5–4 MPa), fracture penetration of interlayers becomes more difficult, and propagation tends to occur within the layers,^39^ forming longer fracture networks. However, the growth of fracture length is suppressed to some extent, with No. 11# seam fracture length increasing by 5.12%, No. 12# by 2.59%, and No. 13# by 6.07%.

The nonuniform propagation coefficient is an indicator used to quantify the degree of difference in fracture length growth across layers during multilayer coal seam fracturing, reflecting the balance (or competitiveness) of fracture propagation in different coal seams. A larger value indicates more significant differences in fracture propagation between layers and more intense competition.

16 $$\xi =\frac{\sigma }{\mu }\times 100\%$$

Where: ξ – nonuniform propagation coefficient; σ – standard deviation of fracture length growth rates across all coal seams; μ – arithmetic mean of fracture length growth rates across all coal seams.

The nonuniform propagation coefficient ξ quantifies the degree of imbalance in fracture length growth across multiple coal seams during hydraulic fracturing. Physically, it reflects the competitive intensity among layers for fluid intake and fracture extension. A higher ξ indicates greater disparity in fracture development between layers, implying that some coal seams are preferentially stimulated while others remain under-stimulated. From an engineering perspective, ξ serves as an indicator of stimulation uniformity: low ξ values (e.g., < 0.1) suggest balanced fracture growth and effective reservoir contact across all layers, whereas high ξ values (e.g., > 0.3) signal potential risks of inadequate stimulation in certain seams, which may lead to uneven gas production and reduced overall recovery efficiency. Therefore, ξ can be used as a diagnostic tool to evaluate fracturing performance and guide optimization strategies such as adjusting injection rates, perforation design, or layer sequencing to achieve more uniform stimulation.

Calculations yield a nonuniform propagation coefficient of 0.33 at this stage (compared to 0.06 under low stress difference 1.5–2 MPa), indicating that high stress difference significantly aggravates the nonuniformity of fracture propagation across layers (competition intensifies).

The relationship between flow proportion and fracture length change is shown in Figure 10. When the interlayer stress difference increases from 1.5 to 4 MPa, the flow proportion of No. 11# coal seam increases from 43 to 73%, No. 12# decreases from 27 to 14%, and No. 13# decreases from 20 to 10%. This indicates that interlayer stress difference directly affects flow allocation among layers by altering fracture propagation paths; increasing stress difference gives thick seams an advantage in flow allocation.

**Figure 10:**
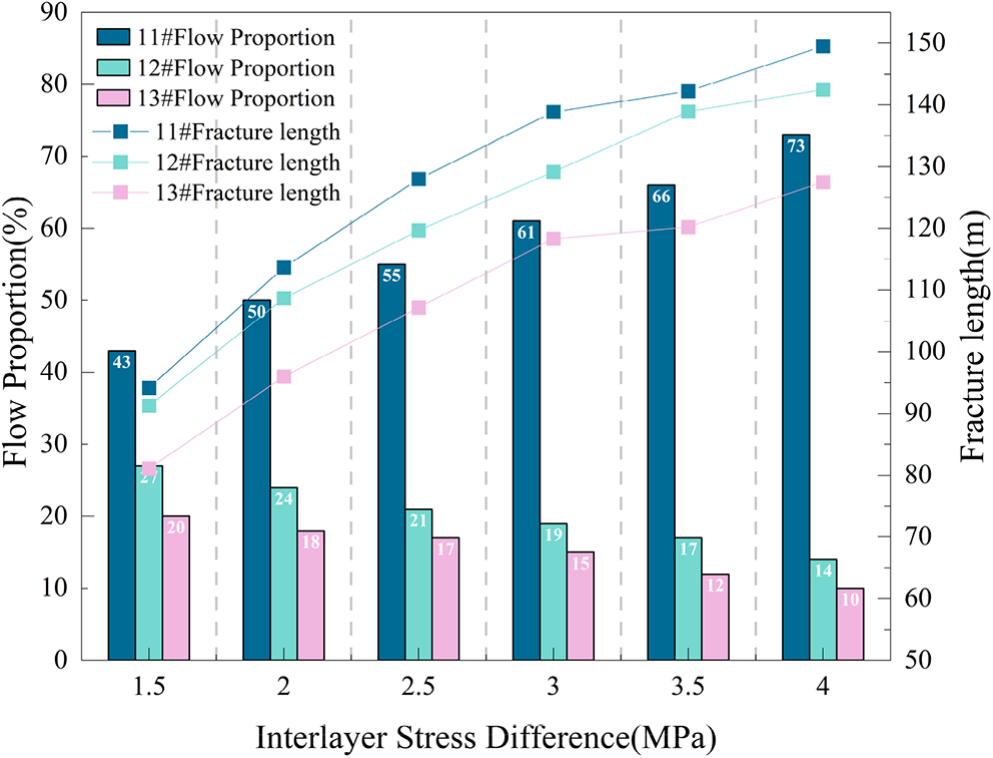
Relationship between flow proportion and fracture length change under different interlayer stress differences.

Figure 11 shows the variation of fluid pressure with step number for No. 11#, No. 12#, and No. 13# coal seams under different interlayer stress differences (5000–20,000 steps, data recorded every 10 steps). The figure shows that at the same time step, a larger interlayer stress difference leads to a faster fluid pressure decline rate but a higher final stable pressure value. The injection volume is kept constant in the simulation, and the fluid volume is adjusted dynamically with time steps. This clearly reflects the three stages of fracture propagation: initiation, extension and equilibrium. During the initial injection phase, fluid pressure rises rapidly until reaching the fracture pressure. Subsequently, as the fracture network expands and fluid loss occurs, pressure enters a phase of dynamic fluctuation.For different coal seams under the same stress difference, the trend of fluid pressure change with step number is similar, but specific values differ due to seam variations.

**Figure 11:**
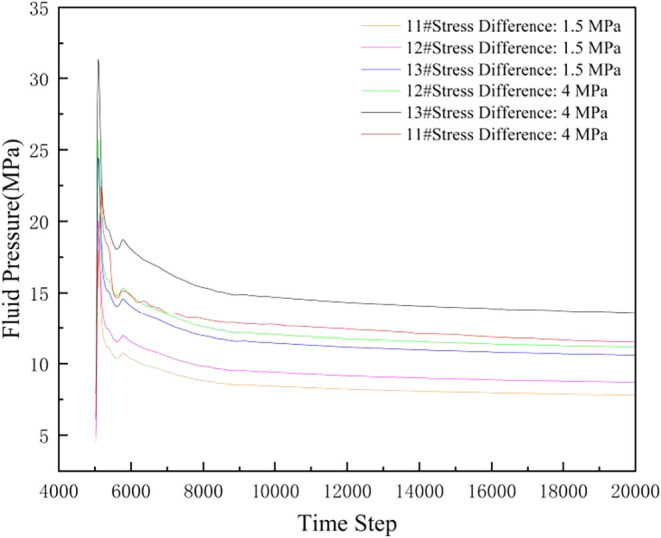
Fluid pressure changes under different interlayer stress differences.

### 4.2 Leakoff Coefficient

Figure 12 shows the influence of the coal seam leakoff coefficient (0.0005, 0.0010, 0.0015, 0.0020, 0.0025, and 0.0030 m/min^0.5^) on fracture propagation. As the coal seam leakoff coefficient gradually increases, fracture length gradually decreases. At low leakoff coefficients (e.g., 0.0005 m/min^0.5^), fluid leakoff is low, fracture internal pressure is high, fracture length decreases uniformly, and morphology is stable. At high leakoff coefficients (e.g., 0.0030 m/min^0.5^), significant fluid leakoff causes a sharp drop in fracture internal pressure,^40^ markedly inhibiting fracture length extension (No. 13# fracture length decreased by 36.70%).

**Figure 12:**
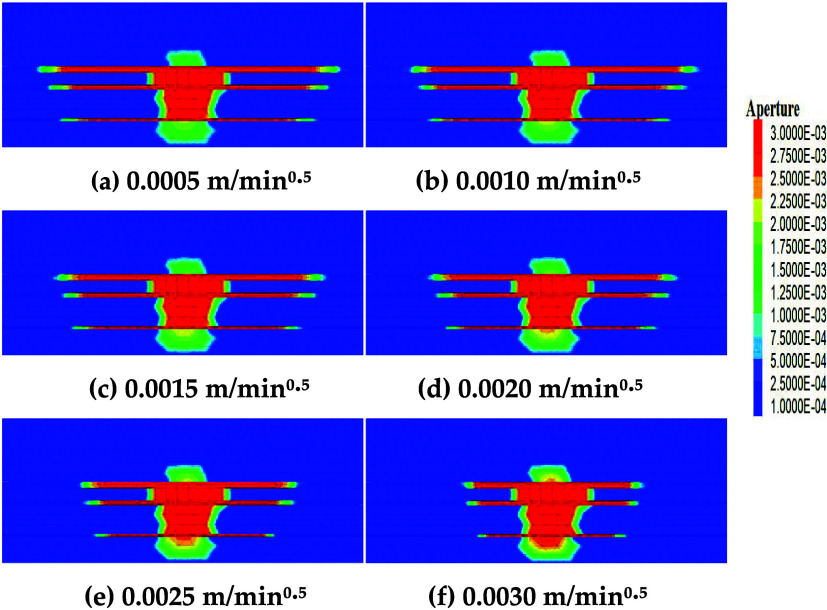
Fracture propagation under different leakoff coefficients (a–f).

The relationship between flow proportion and fracture length change is shown in Figure 13. When the coal seam leakoff coefficient increases from 0.0005 to 0.0015 m/min^0.5^, the fracture length of No. 11# layer decreases by 10.55%, No. 12# by 11.54%, and No. 13# by 8.23%. When the leakoff coefficient increases from 0.0020 to 0.0030 m/min^0.5^, the fracture length of No. 11# layer decreases by 31.65%, No. 12# by 25.95%, and No. 13# by 36.70%. The nonuniform propagation coefficient is 0.16 (compared to 0.12 under low leakoff 0.0005 m/min^0.5^), indicating that under high leakoff conditions, the propagation of thin seams (No. 13#) is more suppressed, and nonuniformity slightly increases.

**Figure 13:**
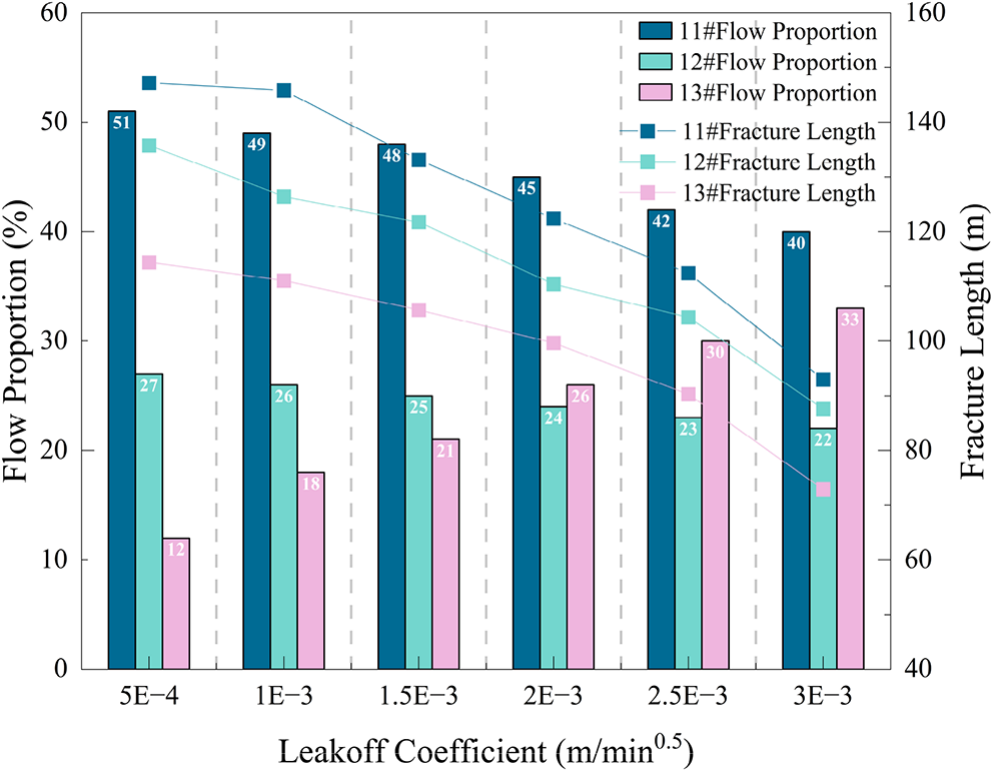
Relationship between flow proportion and fracture length change under different leakoff coefficients.

When the leakoff coefficient increases from 0.0005 to 0.0030 m/min^0.5^, the flow proportion of No. 11# layer decreases from 51 to 40%, No. 12# from 27 to 22%, and No. 13# increases from 12 to 33%. This indicates that the leakoff coefficient affects fluid allocation among layers. As the coal seam leakoff coefficient increases, the flow proportion of thin seams increases, suggesting that high leakoff coefficients are more favorable for thin seams in flow competition.

Figure 14 shows the variation of coal seam pore fluid pressure with step number under different leakoff coefficients. As the coal seam leakoff coefficient increases, coal seam pore fluid pressure decreases. Since the permeability of each layer is similar, thickness plays a dominant role in the ranking of fluid pressure magnitude among layers. Due to the greater thickness of No. 11# layer (3.5 m), it can store more fluid; under the same geological conditions and leakoff, No. 11# layer may have relatively higher pore fluid pressure. Overall pore fluid pressure follows: No. 11# > No. 12# > No. 13#.

**Figure 14:**
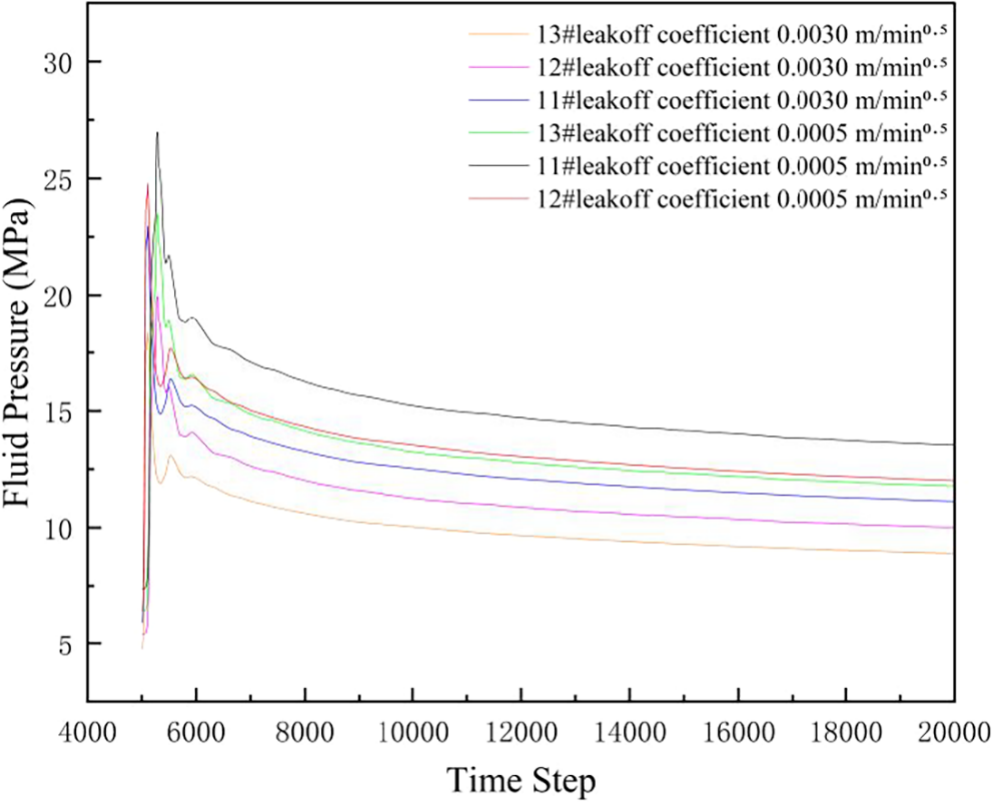
Pore fluid pressure changes under different leakoff coefficients.

### 4.3 Coal Seam Thickness

The thicknesses of the original model’s three coal seams were proportionally scaled down and up by factors of 0.5, 0.8, 1, 1.2, and 1.5 to study fracture competitive propagation laws under different thicknesses. Simulation results are shown in Figure 15. When the thickness is scaled to 0.5 times the original, fracture lengths decrease in all coal seams: No. 11# decreases by 43.01%, No. 12# by 48.01%, and No. 13# by 65.52%. This is mainly because reduced coal seam thickness allows significant fracturing fluid to enter the roof and floor strata, leading to decreased hydraulic fracture length within the coal seams. At 1.5 times the original thickness, No. 11# fracture length increases by 29.65%, No. 12# by 32.99%, and No. 13# by 19.10%. This is primarily due to increased coal seam thickness, which causes significant fracturing fluid influx into the coal seams, inhibiting fluid entry into the higher-strength roof and floor strata. The calculated nonuniform propagation coefficient is 0.28 (0.27 at 0.5 times thickness proportion), indicating that the advantage of thick seams (e.g., No. 11#) expands, leading to persistent stimulation nonuniformity.

**Figure 15:**
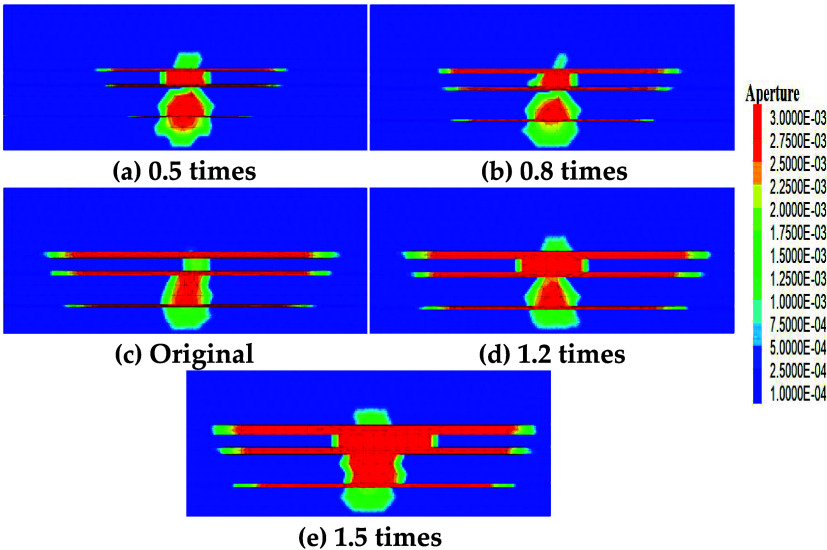
Fracture propagation under different thickness proportions (a–e).

The relationship between flow proportion and fracture length change is shown in Figure 16. As the thickness proportion increases from 0.5 to 1.5, the flow proportion of No. 11# coal seam increases from 38 to 60%, No. 12# decreases from 35 to 18%, and No. 13# decreases significantly from 24 to 13%. This indicates that changes in fracturing layer thickness cause significant shifts in flow proportion among layers. The fluid absorption capacity of thick seams (No. 11#) significantly strengthens, and its flow proportion increases substantially, indicating it is easier to obtain fluid during fracturing, giving it an advantage in flow competition.

**Figure 16:**
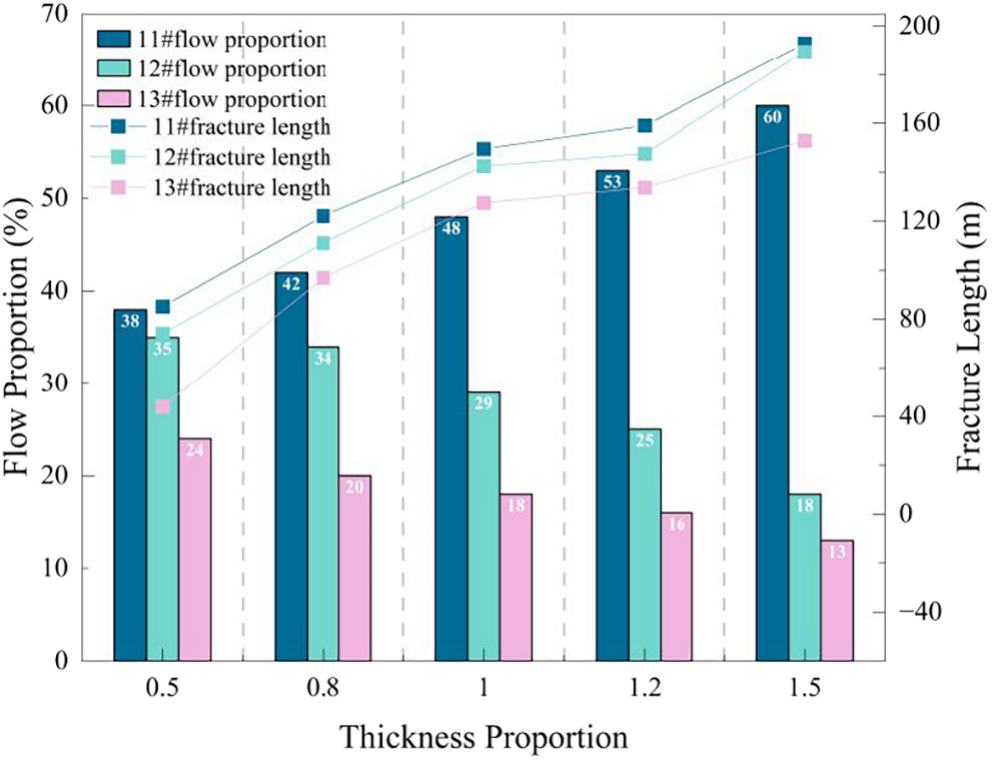
Relationship between flow proportion and fracture length change under different thickness proportions.

From Figure 17, the relationship between thickness proportion and fluid pressure can be clearly observed: In the initial stage of fracturing, fluid pressure in all layers drops rapidly. At this stage, layers with a thickness proportion of 0.5 (No. 11#, 12#, 13#) have relatively lower fluid pressure peaks. This may be because in thinner coal seams during the initial fracturing stage, fluid more easily enters the roof and floor strata, reducing fluid pressure within the coal seams. As fracturing progresses, the overall fluid pressure level of layers with a thickness proportion of 0.5 gradually exceeds that of layers with a thickness proportion of 1.5. This is because thinner coal seams, after experiencing the initial rapid pressure change, gradually tend toward a relatively stable but overall higher-pressure internal fluid flow state. In contrast, thicker coal seams (thickness proportion 1.5) also experience relatively greater leakoff. Although their initial pressure change is relatively gentle, as the step number advances, their pressure drops rapidly due to leakoff, causing fluid pressure to continuously decrease. Ultimately, this results in an overall higher fluid pressure in layers with a thickness proportion of 0.5. Coal seams with smaller thickness proportions have lower pressure peaks in the initial stage, but their overall pressure level exceeds that of seams with larger thickness proportions as step number increases.

**Figure 17:**
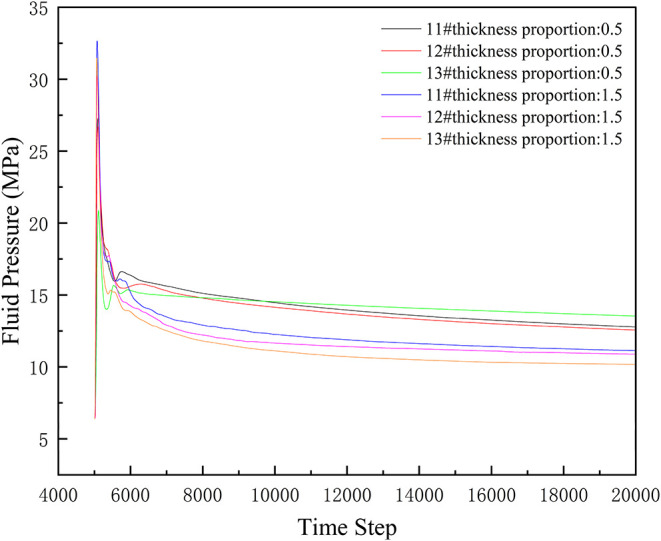
Fluid pressure changes under different thickness proportions.

### 4.4 Fracturing Layer Spacing

Fracturing layer spacing, as a key parameter of multithin-layer reservoir structure, significantly impacts fracture length extension and flow proportion. Hydraulic fracture competitive propagation models were established with spacing proportion factors of 0.5, 0.8, 1, 1.2, and 1.5 relative to the original model spacing. As shown in Figure 18, when fracturing layer spacing is small, in situ stress interference between adjacent layers intensifies, easily suppressing fracture propagation and limiting length growth.This phenomenon is consistent with broader observations of ″fracture-driven interactions″ (FDI or frac hits) in unconventional reservoir development, where tighter spacing and completion designs are critical for managing interwell interference and optimizing production.^41^ At this time, fractures may tend to propagate into the roof and floor strata rather than deep into the coal seams. When layer spacing increases, interlayer stress interference weakens, allowing fractures to propagate more freely in the length direction, which is more conducive to length growth. However, at a spacing proportion factor of 1.2, the interlayer communication area decreases. At a spacing proportion factor of 1.5, communication between No. 12# and No. 13# coal seams fails (spacing reaches 24m). Excessively large layer spacing may disperse fracture propagation energy, hindering effective length growth.

**Figure 18:**
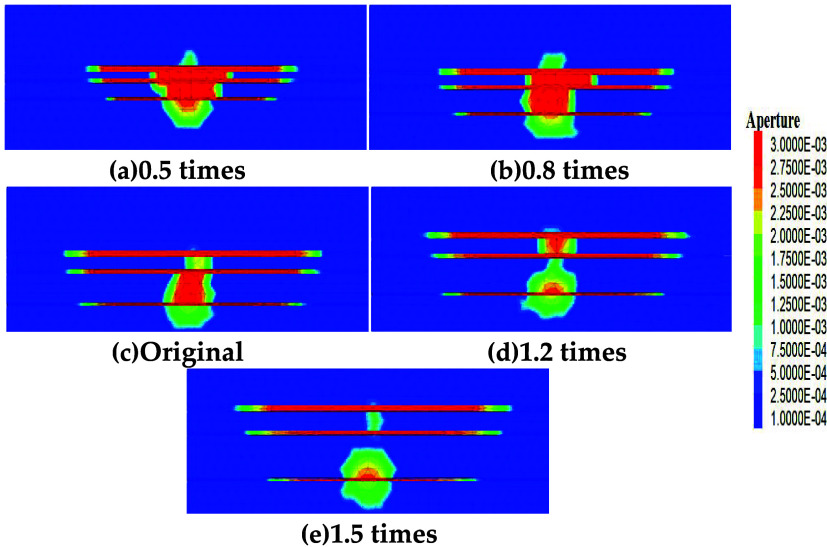
Fracture propagation under different spacing proportions (a–e).

Figure 19 shows the flow proportion and fracture length change for each layer under different spacing proportions. At a spacing proportion of 0.5, No. 13# fracture length decreases by 17.59%, No. 12# by 16.83%, and No. 11# by 20.09%. At a spacing proportion of 1.5, No. 13# fracture length increases by 4.09%, No. 12# by 6.93%, and No. 11# by 7.94%. The calculated nonuniform propagation coefficient is 0.38 (0.10 at 0.5 times spacing proportion), indicating that when layer spacing is too large, differences in fracture propagation across layers significantly increase (e.g., No. 13# propagation is weakest), and the competition pattern tends to solidify.

**Figure 19:**
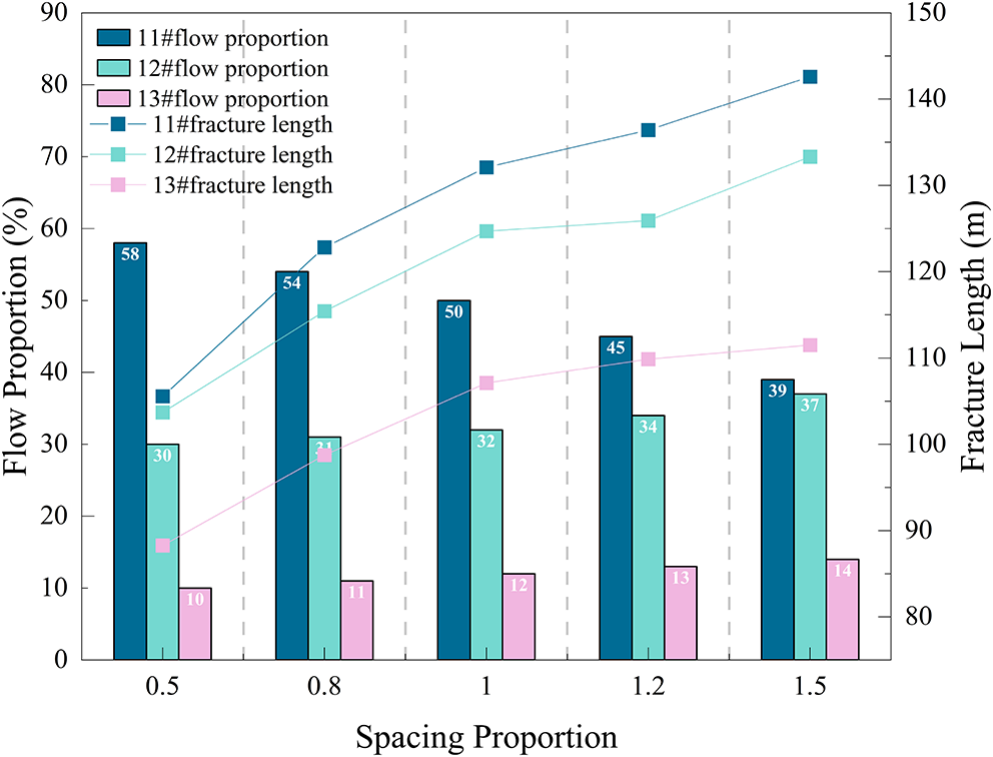
Relationship between flow proportion and fracture length change under different spacing proportions.

As the spacing proportion increases from 0.5 to 1.5, the flow proportion of No. 11# coal seam decreases from 58 to 39%, No. 12# increases from 30 to 37%, and No. 13# increases from 10 to 14%. This indicates that changes in layer spacing alter fracture propagation paths and interlayer communication, thereby affecting the flow allocation proportion among layers. Increasing spacing benefits thin seams in competing for flow, but thick seams still dominate.

Figure 20 shows the fluid pressure curves for No. 11#, 12#, and 13# layers under 0.5 and 1.5 spacing proportions. Fluid pressure in each coal seam exhibits significant differences in the initial stage. As step number increases, pressure gradually stabilizes. Overall, No. 11# layer generally has relatively higher fluid pressure, followed by No. 12#, and No. 13# is relatively lower.

**Figure 20:**
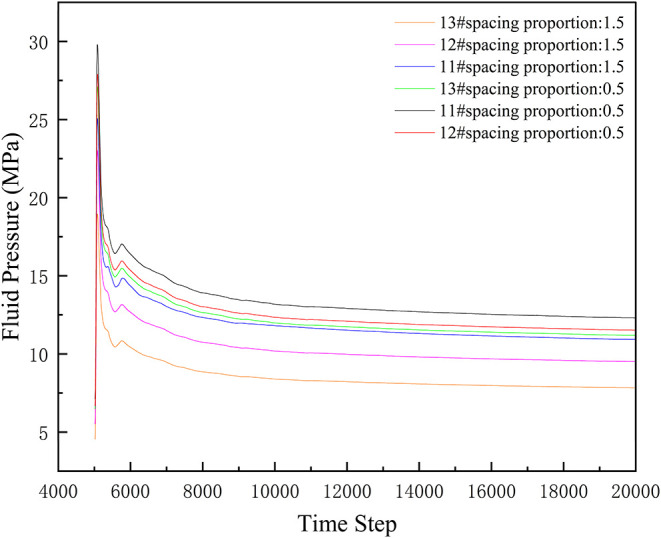
Fluid pressure changes under different spacing proportions.

For the 0.5 spacing proportion, fluid pressure in each coal seam changes rapidly in the initial stage and stabilizes relatively quickly thereafter. This suggests that at smaller spacing, the interaction between coal seams is stronger, fluid competition is more intense, and pressures in each layer adjust rapidly to reach a relatively balanced state.

Under the 1.5 spacing proportion, fluid pressure changes are relatively gentler, requiring more steps to reach a stable state. This indicates that larger spacing weakens the interaction between coal seams, reduces the intensity of fluid competition, slows down the pressure adjustment speed in each layer, and makes them more inclined to maintain their original pressure characteristics.

## 5 Conclusions

(1) At low stress difference (1.5–2 MPa), fractures can easily penetrate interlayers, and fracture length growth across coal seams is relatively balanced. Under high stress difference (3.5–4 MPa), fractures struggle to penetrate interlayers, and the nonuniform propagation coefficient rises to 0.33. Increasing leakoff coefficient significantly reduces fracture length, with thin seams more suppressed; the nonuniform propagation coefficient rises to 0.16, indicating slightly intensified competition. Increasing coal seam thickness promotes fracture length extension but expands the advantage of thick seams; the nonuniform propagation coefficient remains at 0.28. Excessively small layer spacing suppresses fracture length due to stress interference; the nonuniform propagation coefficient is low (0.10). Excessively large spacing (e.g., 24 m) prevents interlayer communication, and the nonuniform propagation coefficient rises to 0.38.
(2) Increasing interlayer stress difference (from 1.5 to 4 MPa) strengthens the dominance of thick seams (No. 11# flow proportion from 43 to 73%). High leakoff coefficient (from 0.0005 to 0.0030 m/min^0.5^) benefits the competitiveness of thin seams (No. 13# proportion from 12 to 33%). Increasing thickness proportion (from 0.5 to 1.5 times) expands the competitive advantage of thick seams (No. 11# proportion from 38 to 61%). Increasing layer spacing (from 0.5 to 1.5 times) improves the competitive environment for thin seams (No. 12# proportion from 30 to 37%).
(3) The elastic modulus of coal seams (5–12 GPa) is significantly lower than that of roof/floor strata (25 GPa), and the leakoff coefficient (5 × 10^–4^m/min^0.5^) is much higher than that of roof/floor strata (1 × 10^–6^m/min^0.5^), causing fractures to primarily extend within coal seams, with significant barrier effects from roof and floor (coal seam flow proportion reaches 90–98%). When the thickness proportion is significantly reduced to 0.5 times, the flow proportion of the thick seam (No. 11#) decreases to 38%. Increasing the layer spacing proportion (1.5 times) weakens the role of roof/floor strata, allows freer fracture propagation within coal seams, and further tilts fluid allocation toward the coal seams.
(4) The competitive propagation mechanisms of fractures revealed in this study exert a direct impact on ultimate well productivity and the economic efficiency of fracturing operations. Nonuniform propagation and dynamic flow allocation jointly govern the effective stimulated reservoir volume in individual thin coal seams: high nonuniformity (e.g., ξ = 0.33 under high stress contrast) leads to inadequate stimulation of certain seams, reducing gas production and economic returns, whereas optimizing interlayer spacing or utilizing leakoff characteristics to enhance fluid entry into thin seams can improve overall reservoir coverage and productivity. The quantitative relationships among parameters, fracture geometry, and flow allocation established in this study serve as a crucial theoretical basis for subsequent productivity simulation and economic evaluation.
(5) Based on the competitive propagation mechanisms revealed in this study, the following targeted engineering recommendations are proposed for optimizing multithin-coal seam fracturing:
  Interlayer stress difference management: When the stress difference between coal seams and roof/floor exceeds 3.5 MPa, the nonuniform propagation coefficient increases significantly (ξ = 0.33), indicating severe stimulation imbalance. In such cases, it is recommended to adopt separate-layer fracturing or adjust injection sequences to ensure adequate stimulation of all seams. For stress differences below 2 MPa (ξ = 0.06), simultaneous fracturing can be considered to enhance operational efficiency.
  Leakoff coefficient consideration: High leakoff coefficients (e.g., > 0.0020 m/min^0^·^5^) disproportionately suppress fracture growth in thin seams (e.g., No. 13# fracture length reduced by 36.70%). To mitigate this, the use of temporary plugging agents or viscosity-enhanced fracturing fluids is recommended to reduce fluid loss and improve thin-seam stimulation.
  Thickness proportion optimization: Thick seams (e.g., No. 11#) dominate fluid allocation when thickness proportion exceeds 1.2 times the original (flow proportion increases to 60%). To prevent overstimulation of thick seams and under-stimulation of thin seams, engineers should consider layered injection with controlled flow rates or the use of diverting agents to balance fluid distribution.
  Layer spacing design: Excessively small spacing (<0.5 times original) intensifies stress interference and suppresses fracture length growth (No. 11# fracture length reduced by 20.09%), while excessively large spacing (>1.5 times original) may prevent interlayer communication (spacing >24 m leads to failed connectivity between No. 12# and No. 13#). An optimal spacing range (approximately 0.8–1.2 times original) is suggested to balance interlayer connectivity and stress interference.
  Real-time monitoring and adjustment: The nonuniform propagation coefficient ξ can be used as a real-time diagnostic indicator during fracturing. If ξ exceeds 0.25 during treatment, operators should consider adjusting pumping schedules or deploying temporary plugging techniques to rebalance fluid allocation and improve overall stimulation uniformity.
  These recommendations provide practical guidance for field engineers to design and adjust fracturing treatments based on site-specific geological conditions, ultimately enhancing reservoir stimulation effectiveness and economic returns.
(6) Study Limitations: This study employs simplified assumptions in the flow allocation criterion, such as constant fluid viscosity and identical pressure drop across all layers. While effective for highlighting the core mechanisms related to permeability and thickness, these assumptions may underestimate the complexities in real-field operations arising from fluid property variations and differences in perforation friction. Furthermore, the model adopts simplified constant stress boundary conditions to apply the in situ stress field. These conditions can effectively represent far-field constraints but fail to consider the potential dynamic changes of boundaries during fracturing, as well as more complex interboundary interactions. Future models could incorporate more dynamic fluid and wellbore mechanics, as well as investigate the influence of more complex boundary conditions (e.g., elastic boundaries), to enhance practical predictive accuracy. Additionally, the coal seams were modeled as an isotropic material, which does not explicitly account for the mechanical anisotropy induced by the cleat system. While the discrete element method inherently captures the direction-dependent failure along discontinuities, the explicit incorporation of orthotropic mechanical properties for the coal matrix could provide a more refined representation in future work.

In addition, the flow allocation criterion adopted in this study assumes constant fluid viscosity, uniform pressure drop, and identical fracture width across all coal seams. These assumptions were made to isolate the effects of permeability and thickness on competitive fracture propagation. However, in actual field operations, factors such as fluid property variations, nonuniform perforation erosion, and layer-specific friction losses can lead to more complex flow behaviors. Therefore, the results presented here should be regarded as a mechanistic insight into the competitive propagation process rather than a direct quantitative prediction for specific field cases. Future studies should consider relaxing these assumptions to incorporate more realistic fluid and wellbore dynamics, as well as the anisotropic mechanical behavior of coal seams induced by cleat systems.

## Data Availability

The data that supports the findings of this study is available within the article.
